# Analysis of pulsating variable stars using the visibility graph algorithm

**DOI:** 10.1371/journal.pone.0259735

**Published:** 2021-11-17

**Authors:** Víctor Muñoz, N. Elizabeth Garcés

**Affiliations:** Departamento de Física, Facultad de Ciencias, Universidad de Chile, Santiago, Chile; University of Burgundy, FRANCE

## Abstract

We study the light curves of pulsating variable stars using a complex network approach to build visibility graphs. We consider various types of variables stars (e.g., Cepheids, *δ* Scuti, RR Lyrae), build two types of graphs (the normal visibility graph (VG) and the horizontal visibility graph (HVG)), and calculate various metrics for the resulting networks. We find that all networks have a power-law degree distribution for the VG and an exponential distribution for the HVG, suggesting that it is a universal feature, regardless of the pulsation features. Metrics such as the average degree, the clustering coefficient and the transitivity coefficient, can distinguish between some star types. We also observe that the results are not strongly affected by the presence of observation gaps in the light curves. These findings suggest that the visibility graph algorithm may be a useful technique to study variability in stars.

## Introduction

Currently, complex networks are used in various areas, and the number of topics in which they are being useful keeps growing [1–9].

An interesting problem is how to build a complex network from a time series, which is a universal problem, considering that time series is the primary input that basic sciences receive from nature.

Several ways to build these networks have been proposed [10], but there is one which leads to particularly interesting results, called the visibility graph algorithm [11], which takes a time series and maps it into a graph. In this graph, a node corresponds to a given datum in the time series, and two nodes are connected if visibility exists between the corresponding data, *i.e*. if there is a straight line that connects the data, provided that this “visibility line” is always above the data curve.

The VG algorithm has proven to be a useful way to study the structural properties of time series [12–15], capturing their level of regularity or randomness. The primary advantage of this method compared to others is its low computational cost. This method can be used to detect nontrivial properties from the series, such as fractality [11] and reversibility [16].

In this study, we are interested in applying the VG algorithm to the study of variable stars. The input are light curves, which are time series of the luminosity of a star.

Variable stars are stars that change their luminosity in time when viewed from the Earth or satellites. In many cases, stars exhibit this behavior periodically. We study one specific type of variable star known as pulsating stars, where sound waves travel across the stars’ interior, typically making their radii change in time. When their radii increases (decreases), the star becomes colder (hotter). Conversely, this change in temperature leads to a luminosity change due to the Stefan-Boltzmann equation. The best known pulsating stars are the Cepheids, popular due to their period-luminosity relation [17]. These stars show regular changes in their light curves, and their pulsating mechanism is fairly well understood. RR Lyrae is another type of pulsating star, also called “short-period Cepheids”, due to their much smaller pulsating period compared to Cepheids. They also exhibit a period-luminosity relation, which is important in astronomy because it can be used to infer the distance to the star, which is why Cepheids are popularly referred to as standard candles.

Additionally, there is one particular type of pulsating star that has been difficult to understand: *δ* Scuti stars. These stars are also Cepheids, and due to their low mass they are also called dwarf Cepheids. They also exhibit the period-luminosity relation. The difference between the classical Cepheids, RR Lyrae and *δ* Scuti is their pulsation mode. The first two pulsate only in radial modes; however, *δ* Scuti stars pulsate in both radial and non-radial modes, making their light curve difficult to understand. For this reason, several studies have attempted to understand their pulsation mechanism [18, 19], including its possible fractal behavior [20].

In this study, we apply the VG algorithm to the study of light curves of variable stars. We then select a number of characteristic variable stars (Cepheids, RR Lyrae, *δ* Scuti), apply the VG technique to their respective light curves, and discuss to what extent it provides useful information about their variability.

This paper is structured as follows. First, we briefly discuss the primary definitions and general results on visibility graphs and complex networks that we will need in this study. Then, we discuss the data, provide details on how we deal with them for the VG approach, and show the results of various analyses. Finally, we summarize and discuss the results.

## Materials and methods

### Visibility graph

Among various proposals to map a time series to a graph, the proposal of the visibility graph (VG) in [11] is particularly interesting because it has been successfully applied to a large variety of problems in several fields of research [21, 22]. The VG is a geometrical way to build a complex network from a time series, where every data point in a 2D plot is a node, and two nodes are connected if they can be joined by a visibility line; thus, all intermediate data lie below that line.

There are two primary methods of building visibility graphs. For a normal visibility graph (VG), the visibility line joins data points, which allows lines to have different slopes, depending on the relative height of the points. Data points can also be replaced by vertical bars, joining them with the *x*-axis. Then, visibility lines are drawn parallel to the *x*-axis, starting at a data point, until it finds another datum’s bar. This method is called the horizontal visibility graph (HVG) [23].

Note that the resulting graphs are connected (every node has at least one connection to its neighbors) and invariant under affine transformations (translations and rescalings) of the vertical and horizontal axes. Many studies [11, 21, 22, 24] have shown that the VG provides nontrivial information from the time series and that it can distinguish between some types of fractal series [11].

The HVG has turned out to be an interesting variant of the VG. Due to its definition, the number of connections between nodes is lower, which could lead to poorer statistics. However, studies have demonstrated its usefulness in time series. One of the HVG’s interesting features is that some analytical results can be obtained for the metrics of the HVG, at least for simple time series. For example, the HVG is a small-world network with an average degree 〈*k*〉 = 4 and an exponential degree distribution [23] for fully uncorrelated chaotic or correlated stochastic time series.

In general, it can be shown that for all horizontal visibility graphs [23],

$$\begin{array}{c} 2\leq \langle k\rangle \leq 4\;, \end{array}$$ (1)

where the lower bound occurs for constant time series (one forward and one backward connection).

Various studies have been devoted to establishing whether HVG can actually distinguish between deterministic and stochastic time series [23, 25], and to determine the periodicity of time series using the HVG [26].

In this study, we apply the VG and HVG techniques to each selected time series (details in the Variable star data Section). Once the networks are built, we calculate a few metrics to characterize them, including degree (the number of connections of each node, denoted by *k*_*i*_); the average degree:

$$\begin{array}{c} \left\langle k\right\rangle =\frac{1}{N}\sum_{i}k_{i}\;; \end{array}$$ (2)

the clustering coefficient

$$\begin{array}{c} C=\frac{1}{N}\sum_{i=1}^{N}C_{i}\;, \end{array}$$ (3)

where *C*_*i*_ is the clustering coefficient of node *i*, given by:

$$\begin{array}{c} C_{i}=2\frac{\lambda {\left(i\right)}_{G}}{k_{i}\left(k_{i}-1\right)}\;, \end{array}$$ (4)

with λ(*i*)_*G*_ being the number of triangles that contain the node *i*); and the transitivity coefficient:

$$\begin{array}{c} T=\frac{\lambda_{G}}{\nu_{G}}\;, \end{array}$$ (5)

where λ_*G*_ is the total number of triangles in the graph, and *ν*_*G*_ the total number of triplets (subgraphs with three nodes and two edges). These last two metrics quantify the number of triangles in the network (i.e., the probability that, if two nodes *a* and *b* are connected to a third one, then *a* and *b* are also connected to each other).

### Variable stars data

We analyze data for 48 pulsating stars taken from the OGLE-III catalog [27–29]. The selected stars include various types of variable stars, including classical Cepheids, RR Lyrae, and *δ* Scuti, that belong to the Large Magellanic Cloud. Classical Cepheids and RR Lyrae pulsate in radial modes only, while *δ* Scuti exhibit hybrid (both radial and non-radial) pulsations. Also, regarding Cepheids, three types have been considered: stars pulsating in (a) the fundamental mode, (b) the first overtone, and (c) the second overtone.

The OGLE-III catalog has data corresponding to the V (visible) and I (infrared) bands. However, the amount of data is much higher for the I band; thus, we have chosen to limit analysis to this band to achieve better statistical results.

Most infrared light curves have an order of 850 points, with a few of them (e.g., *δ* Scuti stars) having approximately 1200 points.

Table 1 describes each star used in this study and shows the star identification in the OGLE-III catalog, the period *P*_1_ of the first overtone in days, the period *P*_2_ of the second overtone in case it exists, the pulsation mode, and the number of data points in the catalog.

**Table 1:** Stars from the OGLE-III catalog, selected for this study: *δ* Scuti (DSCT), Cepheids (CEP), and RR Lyrae (RRLYR) stars. The oscillation mode can be “Single” (it is not possible to unambiguously identify the pulsation mode [29], “F” (fundamental mode pulsators), “1O” (first overtone pulsators) or “1O/2O” (double-mode pulsators, in first and second overtone). For RR Lyrae stars, mode is given by the type of the star: RRab are fundamental mode pulsators, RRc are first overtone mode pulsators and RRd are double-mode pulsators (in first and second overtone).

Star	*P*_1_ (d)	*P*_2_ (d)	Mode	Data points
OGLE-LMC-DSCT-1402	0.066595049		Single	1015
OGLE-LMC-DSCT-1417	0.134203370		Single	1015
OGLE-LMC-DSCT-1463	0.109205921		Single	1062
OGLE-LMC-DSCT-1475	0.078958472		Single	1067
OGLE-LMC-DSCT-1478	0.099254286		Single	1063
OGLE-LMC-DSCT-1507	0.078819742		Single	1061
OGLE-LMC-CEP-1753	2.5745626		F	1201
OGLE-LMC-CEP-1780	3.8186769		F	883
OGLE-LMC-CEP-1791	3.6612191		F	882
OGLE-LMC-CEP-1794	4.3328495		F	885
OGLE-LMC-CEP-1802	6.0134457		F	883
OGLE-LMC-CEP-1857	4.8203638		F	866
OGLE-LMC-CEP-1769	2.0801626		1O	852
OGLE-LMC-CEP-1838	1.6431880		1O	890
OGLE-LMC-CEP-1839	0.3415128		1O	886
OGLE-LMC-CEP-1860	2.0013048		1O	819
OGLE-LMC-CEP-1867	3.9414981		1O	795
OGLE-LMC-CEP-1868	3.6616334		1O	880
OGLE-LMC-CEP-1708	0.2419824	0.1942195	1O/2O	799
OGLE-LMC-CEP-1710	0.8885263	0.7167444	1O/2O	839
OGLE-LMC-CEP-1734	0.5901103	0.4745227	1O/2O	894
OGLE-LMC-CEP-1823	0.7833440	0.6311927	1O/2O	884
OGLE-LMC-CEP-1869	0.2930049	0.2350355	1O/2O	884
OGLE-LMC-CEP-1934	1.2950682	1.0367763	1O/2O	894
OGLE-LMC-RRLYR-12906	0.4684054		RRab	884
OGLE-LMC-RRLYR-12932	0.7102158		RRab	882
OGLE-LMC-RRLYR-12994	0.6875818		RRab	882
OGLE-LMC-RRLYR-13000	0.5694727		RRab	885
OGLE-LMC-RRLYR-13030	0.5246726		RRab	883
OGLE-LMC-RRLYR-13125	0.8967681		RRab	1194
OGLE-LMC-RRLYR-12737	0.3445523		RRc	769
OGLE-LMC-RRLYR-12756	0.3383183		RRc	797
OGLE-LMC-RRLYR-12762	0.3452761		RRc	801
OGLE-LMC-RRLYR-13096	0.3194882		RRc	1249
OGLE-LMC-RRLYR-13148	0.3106766		RRc	825
OGLE-LMC-RRLYR-13185	0.3049260		RRc	1302
OGLE-LMC-RRLYR-13369	0.3603041	0.4839633	RRd	875
OGLE-LMC-RRLYR-13783	0.3501258	0.4711385	RRd	879
OGLE-LMC-RRLYR-13960	0.3463834	0.4661516	RRd	881
OGLE-LMC-RRLYR-14015	0.3528262	0.4745833	RRd	880
OGLE-LMC-RRLYR-14162	0.3611600	0.4853301	RRd	745
OGLE-LMC-RRLYR-14519	0.3457231	0.4652083	RRd	879

**Fig 1:**
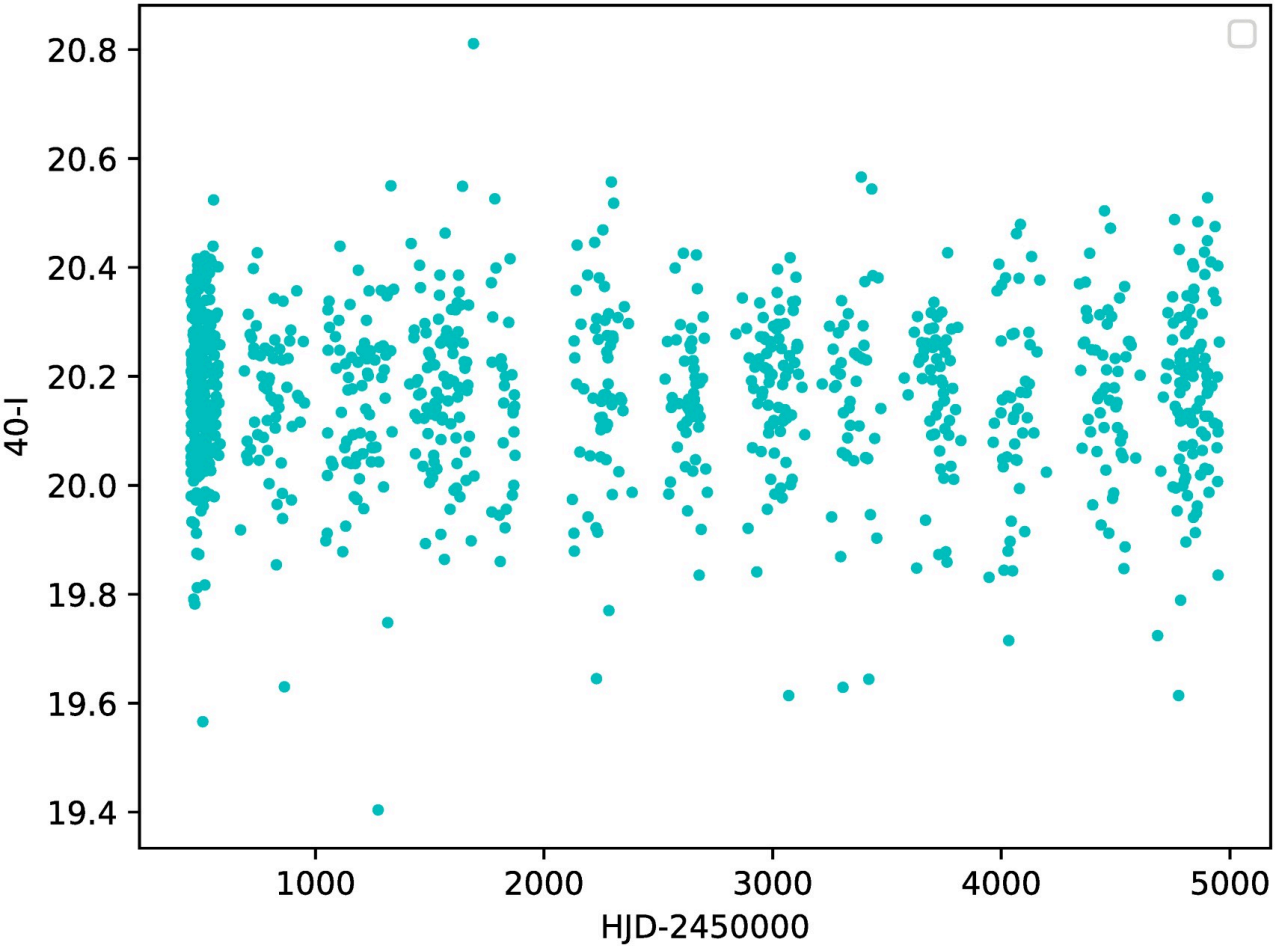
Light curve for the *δ* Scuti star OGLE-LMC-DSCT-1402.

Fig 1 shows a typical light curve obtained from the catalog for a *δ* Scuti star as an example. The first relevant observation for the purposes of this study is the uneven spacing of data, and the existence of evident gaps.

It is common to have unevenly spaced light curves, as various situations may affect Earth-based observations: they can only be made at night, or may be blocked by other objects such as the Moon, or can be limited to a certain season during the year. Also, data collection can be interrupted by technical problems or observation schedule issues.

Thus, we face an inherent issue with all light curves that we intend to analyze. Because the VG involves a geometric rule to map the time series into a network, any choice we make to mitigate this issue will affect the resulting network, its metrics, and the analysis.

Thus, we use three strategies to establish to what extent the strategies themselves affect the conclusions. First, we consider the full time series, including whatever gaps are present, thus ignoring the fact that there are missing data (Full time series Section). Second, we split the time series into subsets that are separated by the gaps and consider each subset as a time series in itself. Then, we obtain a graph and its metrics for each subset, and average the results to obtain a result representative of the entire time series (Observation windows Section). Finally, we consider the phased time series, which is constructed by adding the information about the pulsation period *T* of the star, and then plotting the light curve not versus time but versus time modulo *T* (Phased light curves Section). The phased curve is a common way to represent the light curve of stars but is not an affine transformation of the plot and will thus modify the VG and its metrics. By taking the three strategies mentioned, we expect to understand to what extent this is relevant.

The definition of HVG implies that it is the same for an evenly or an unevenly spaced time series (if the data sequence is the same); thus, we can expect that the HVG will be less sensitive to the existence of gaps than the VG.

## Results

We now show the results obtained when the VG and HVG techniques are applied to the light curves of the selected stars, for each of the three strategies to deal with the gaps.

### Full time series

As mentioned in the Variable star data section, the light curves have different lengths: most have about 850 points, while the *δ* Scuti stars have approximately 1200. Considering size effects, we have studied the *δ* Scuti time series both with all of the data and by truncating it to the first 850 points.

We have calculated the degree distribution for each selected star, and for both the VG and HVG.

**Fig 2:**
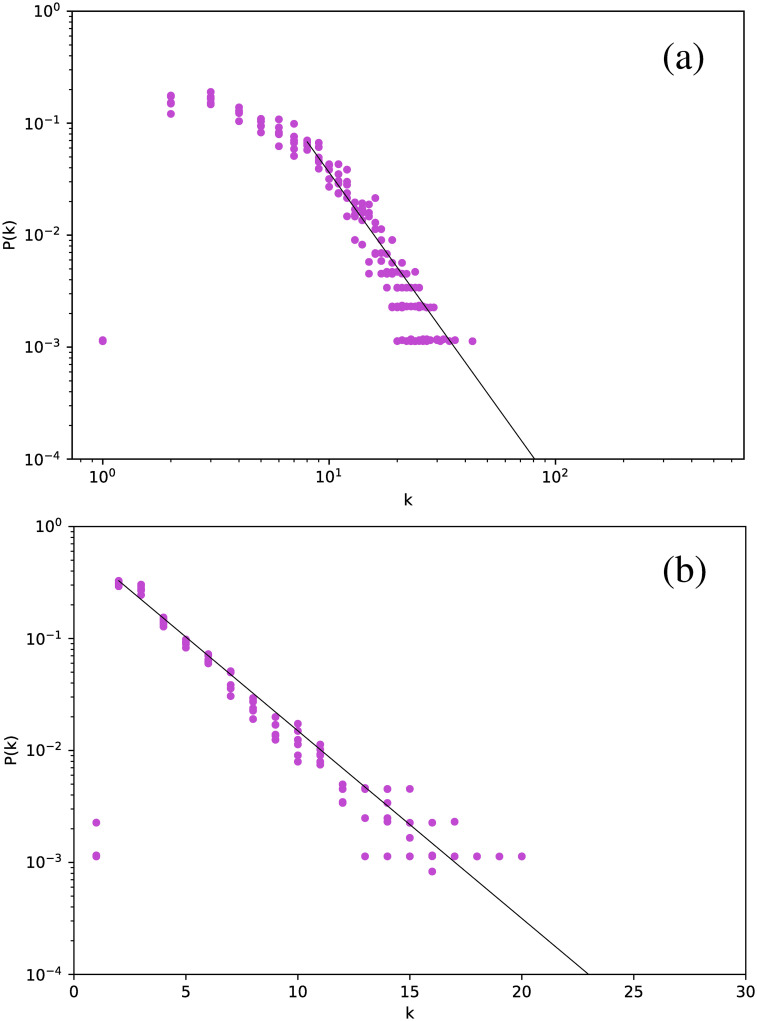
Degree distribution for the Cepheids pulsating in the fundamental mode, for networks built for the full time series. (a) Visibility Graph, log-log plot. (b) Horizontal Visibility Graph, semilog plot.

Fig 2 shows the degree distribution for the Cepheids pulsating in the fundamental mode. All stars have been included in the same plot.

The first interesting result is that, essentially, all stars yield essentially the same degree distribution. However, this distribution is scale-free for the normal VG and exponential for the HVG. As we will see below, this turns out to be a robust result for all types of stars studied. In this case, the curves are well fitted by a power-law exponent *γ*_VG_ ∼ 2.82, and an exponential decay exponent *γ*_HVG_ ∼ 0.39, respectively.

**Fig 3:**
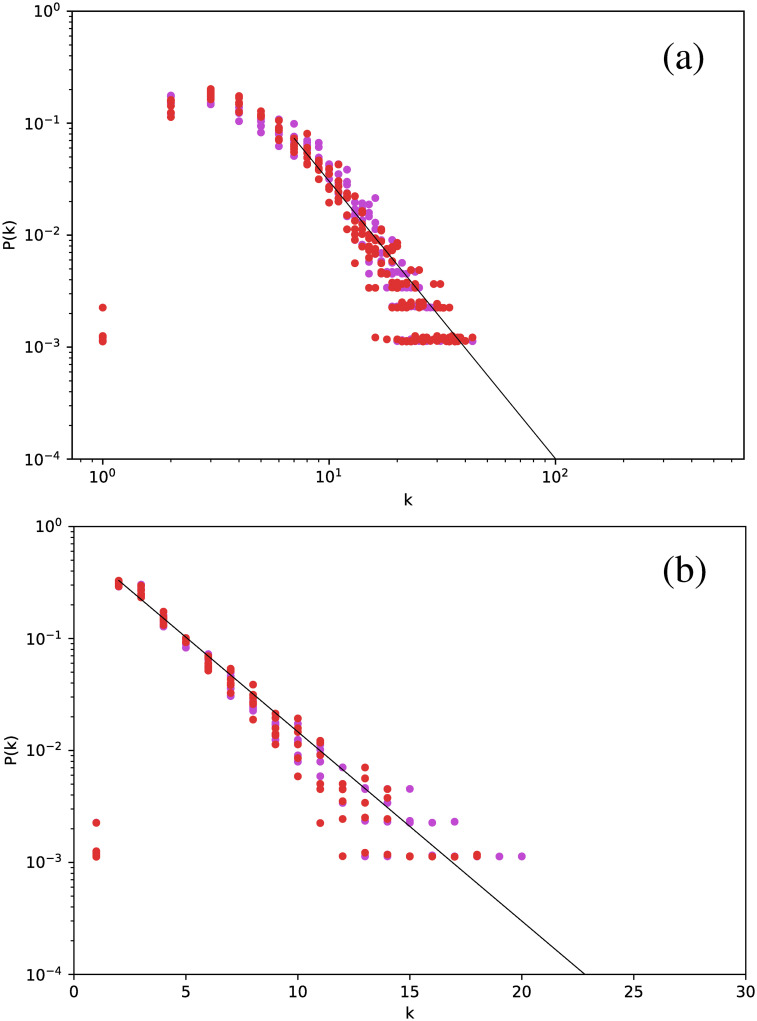
Same as Fig 2, but for all Cepheids, including those pulsating in the fundamental mode (purple dots), the first overtone (orange dots), and the second overtone (red dots).

If a similar analysis is made for the Cepheids pulsating in their first or second overtone, we obtain similar results. We show this in Fig 3, where results for all Cepheids are included in the same plot. Thus, all Cepheids lead to networks with the same degree distribution.

**Fig 4:**
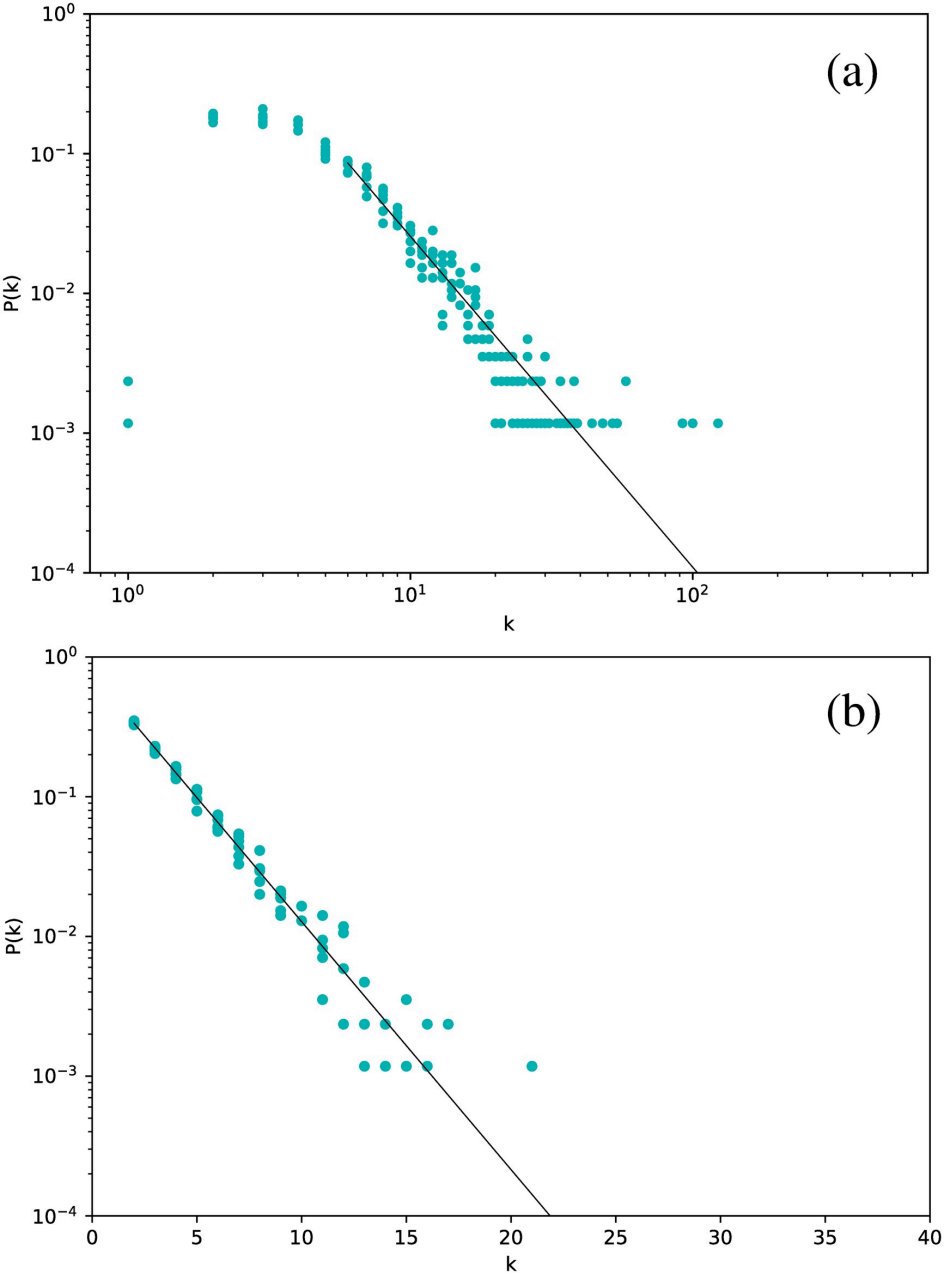
Same as Fig 2, but for *δ* Scuti stars.

If we now consider *δ* Scuti stars, Fig 4 is obtained. The structure is exactly the same: all stars follow the same distribution, which is scale-free for the VG and exponential for the HVG. However, we notice a marginal difference in slope with respect to the Cepheids, namely *γ*_VG_ = 2.37 and *γ*_HVG_ = 0.41.

**Fig 5:**
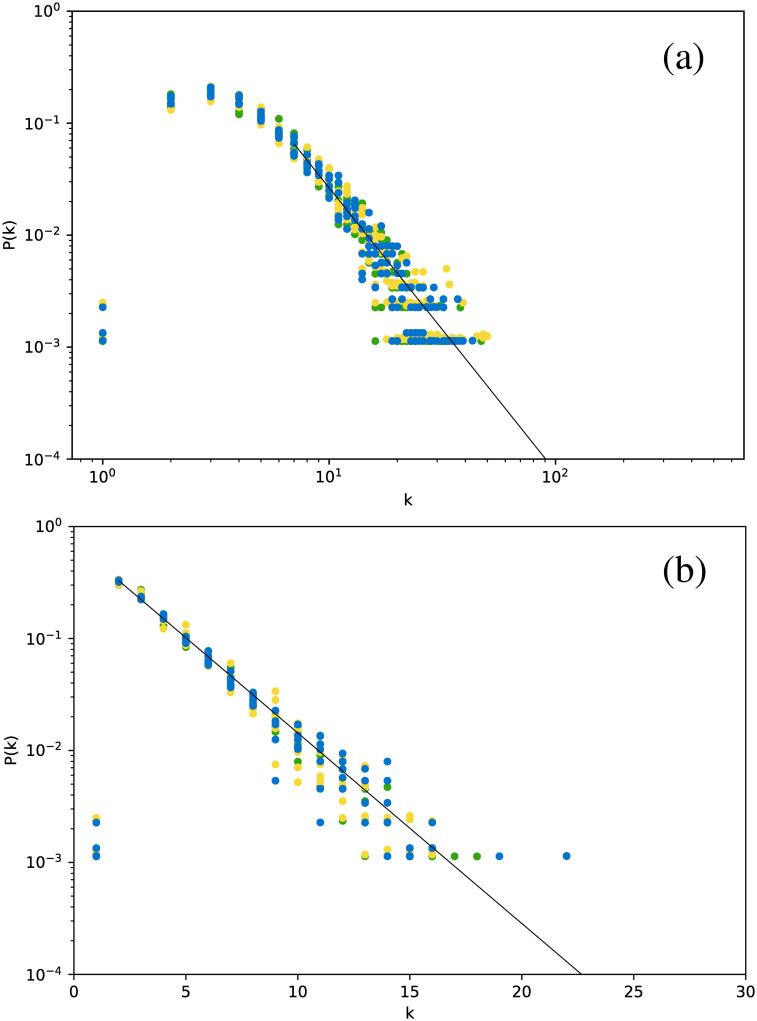
Same as Fig 2, but for all RR Lyrae stars.

Analogous results are obtained for RR Lyrae stars, with characteristic exponents that are more similar to Cepheids than to *δ* Scuti (Fig 5).

Thus, the VG and HVG techniques reveal a universal behavior of the light curves of variable stars, regardless of the nature of the oscillation, including the presence of overtones or of non-radial modes.

These results are interesting when one accounts for the shape of the respective light curves. In particular, for RR Lyrae RRc, the shape of the light curves are much more sinusoidal compared to the others, which resemble a seesaw curve. However, this feature is not relevant for the qualitative behavior of the degree distribution. When compared to artificial time series, the results obtained are similar to those given by fractal time series in the case of VG [11] and to Gaussian correlated or noisy periodic time series in the case of HVG [30].

However, unlike such examples, the light curves in this study are periodic time series. In this sense, the initial guess is that the fluctuation level in the light curves is sufficient to yield a network whose degree distribution resembles a random time series.

Table 2 shows the exponents obtained for each star type, both for the HVG (exponential fit) and the VG (power-law fit).

**Table 2:** Exponents for the degree probability distribution, where *γ*_HVG_ is the exponent for an exponential fit of the HVG distribution and *γ*_VG_ for a power-law fit of the VG distribution.

Star Type	*γ*_HVG_	*γ*_VG_
*δ* Scuti	0.408 ± 0.003	2.59 ± 0.02
*δ* Scuti (t)^1^	0.409 ± 0.003	2.37 ± 0.01
Cepheids F	0.386 ± 0.007	2.82 ± 0.02
Cepheids 1O	0.392 ± 0.005	2.52 ± 0.02
Cepheids 1O/2O	0.388 ± 0.003	2.69 ± 0.02
RR Lyrae RRab	0.390 ± 0.004	2.43 ± 0.01
RR Lyrae RRc	0.390 ± 0.004	2.41 ± 0.02
RR Lyrae RRd	0.396 ± 0.002	2.50 ± 0.02

First, size effects are not relevant for *δ* Scuti stars, at least for the HVG. They have the largest number of points, as shown in Table 1, which could make the results incomparable to those of the other types. However, when the time series is truncated to the first 850 data points, which is of the same order as the other stars, the same exponent is found for the HVG. However, the VG result is different, which must be considered during analysis.

Additionally, we notice that all exponents are approximately equal (approximately 0.4 for the HVG and 2.5 for the VG), but marginal differences can be found when comparing different star types. In effect, the HVG yields clearly larger results for *δ* Scuti stars, while it is difficult to discriminate between the other stars. Discrimination is much better for the VG, although values for *δ* Scuti are not larger than the rest unlike with the HVG. With the VG, *δ* Scuti, particularly Cepheids, have larger exponents than RR Lyrae stars.

From these results, it is straightforward to calculate the average degree for each star. We have also calculated two additional metrics: the clustering coefficient and the transitivity coefficient.

Because the results may be affected by size effects, each time series has a different number of points; thus, each network has a different number of nodes and connections. We thus present the results of these metrics as a function of the number *m* of connections of the network for each individual star.

**Fig 6:**
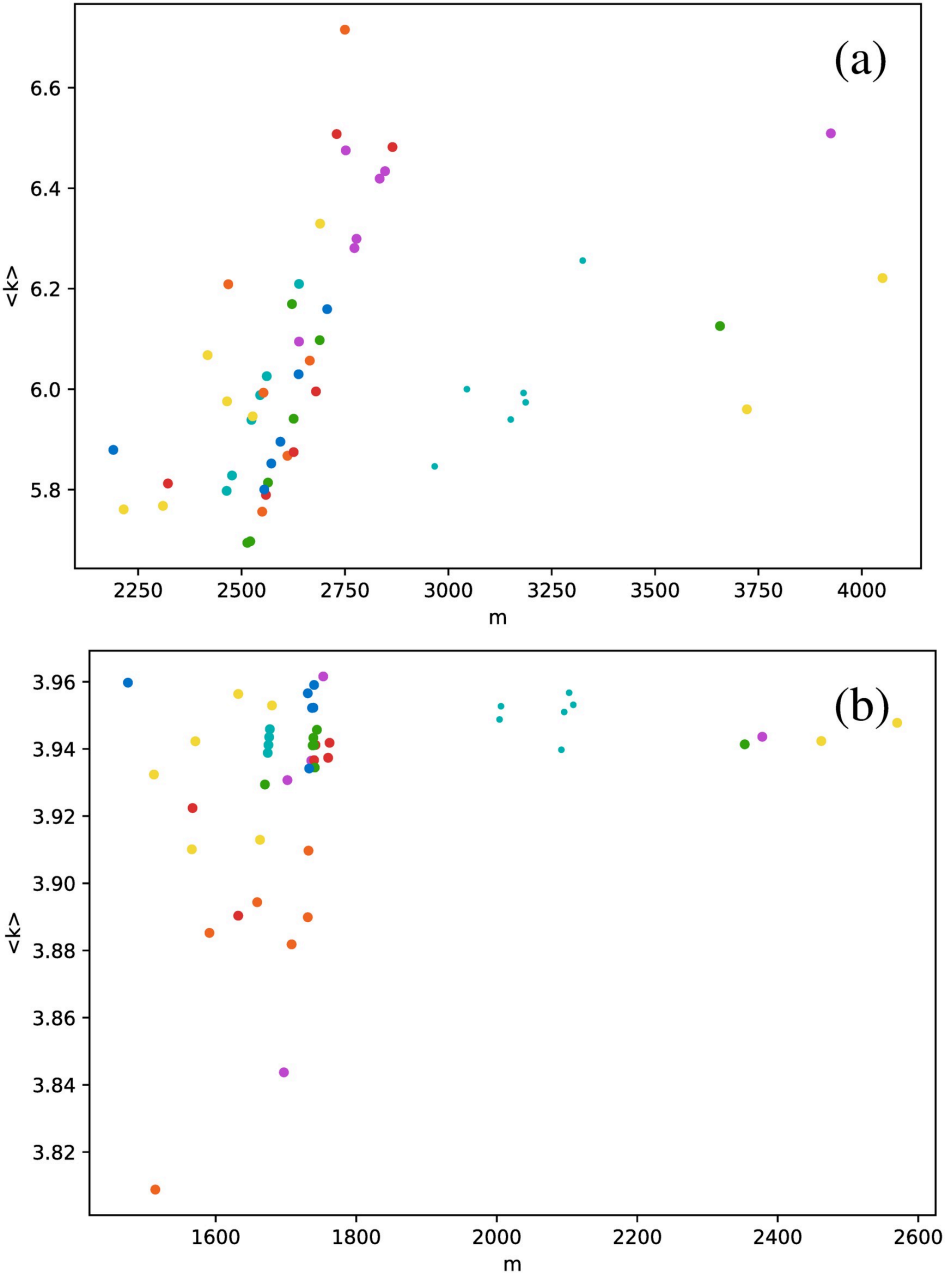
Average degree versus number of connections *m*, for networks built for the full time series. Each dot corresponds to a single star, and each color represents a star type. Magenta: Cepheids pulsating in the fundamental mode; orange: Cepheids pulsating in the first overtone; red: Cepheids pulsating in the second overtone; cyan: delta Scuti (large dots include all data points, small dots include only the truncated series); green: RR Lyrae RRab; yellow: RR Lyrae RRc; blue: RR Lyrae RRd. Each subfigure corresponds to one type of visibility graph: (a) VG, (b) HVG.

Fig 6 shows the average degree as a function of the number of connections.

We first observe that there is no clear correlation between 〈*k*〉 and *m*, except maybe for Cepheids in the second overtone (red dots) and RR Lyrae RRd (blue points), where 〈*k*〉 seems to vary linearly with *m* for the normal VG.

As mentioned in the Visibility graph section, the number of connections in a VG is higher than in the corresponding HVG, and thus the same can be said about 〈*k*〉, and this can be actually observed when comparing Fig 6(a) and 6(b). We also find that the result for the HVG satisfies Eq (1). However, not all star types are distributed equally in the range given by this general equation; thus, it is interesting to discuss Fig 6 in more detail. In effect, both *δ* Scuti (cyan dots) and RR Lyrae RRd (blue dots) are clearly confined to a small range near to 4, suggesting that their respective light curves are more complex than the rest. This result is consistent with the fact that the frequencies of *δ* Scuti are better understood than their modes, which, as mentioned in Table 1, cannot be determined unambiguously, and the fact that RRd stars pulsate in two modes simultaneously, as Cepheids 1O/2O do, but in a less sinusoidal way.

**Fig 7:**
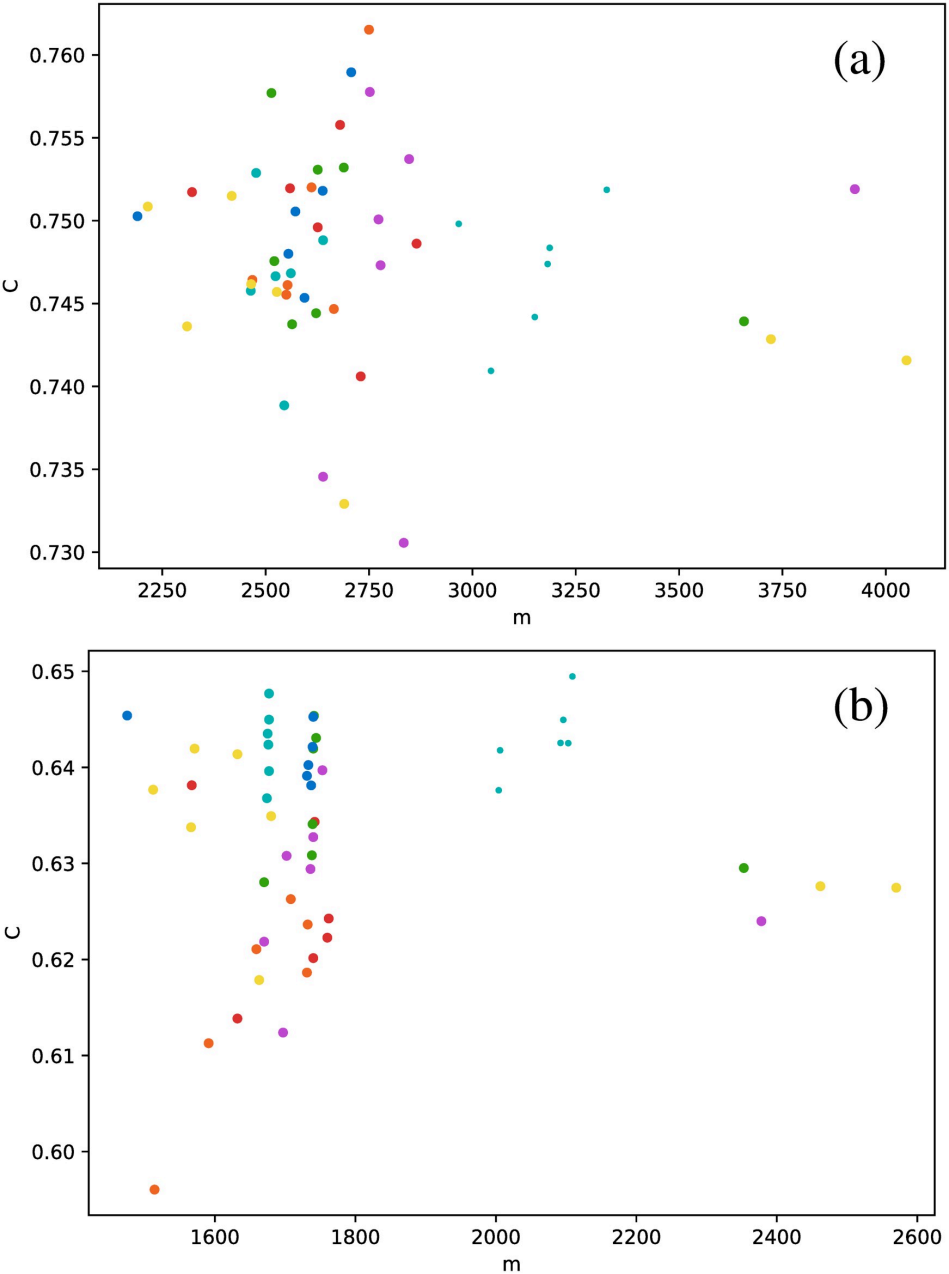
Same as Fig 6, but for the clustering coefficient.

These results suggest that HVG is a better approach to distinguish between star types than the VG, at least regarding average degree. When we study other metrics, analogous conclusions may be drawn, as shown in Fig 7, where the clustering coefficient is plotted as a function of the number of connections.

In general, the clustering coefficient can have any value between 0 (for every node, all of its connections are not connected between them, *e.g*. a star graph), and 1 (for every node, all connections are connected between them, *e.g*. a fully connected network). We notice that, for all stars, networks have rather intermediate values of the clustering coefficient, and confined to a narrow interval. And, as already observed in Fig 6, the HVG has better discrimination capabilities than the VG. *δ* Scuti and RR Lyrae RRd (cyan and blue dots, respectively) are confined to an even smaller range of values of *C*, which is nontrivial, because in general, there is no correlation between the degree of a node and its clustering coefficient, or between their respective average values over the entire network. Thus, there is no *a priori* reason to expect that both types of stars could be distinguished by both metrics.

**Fig 8:**
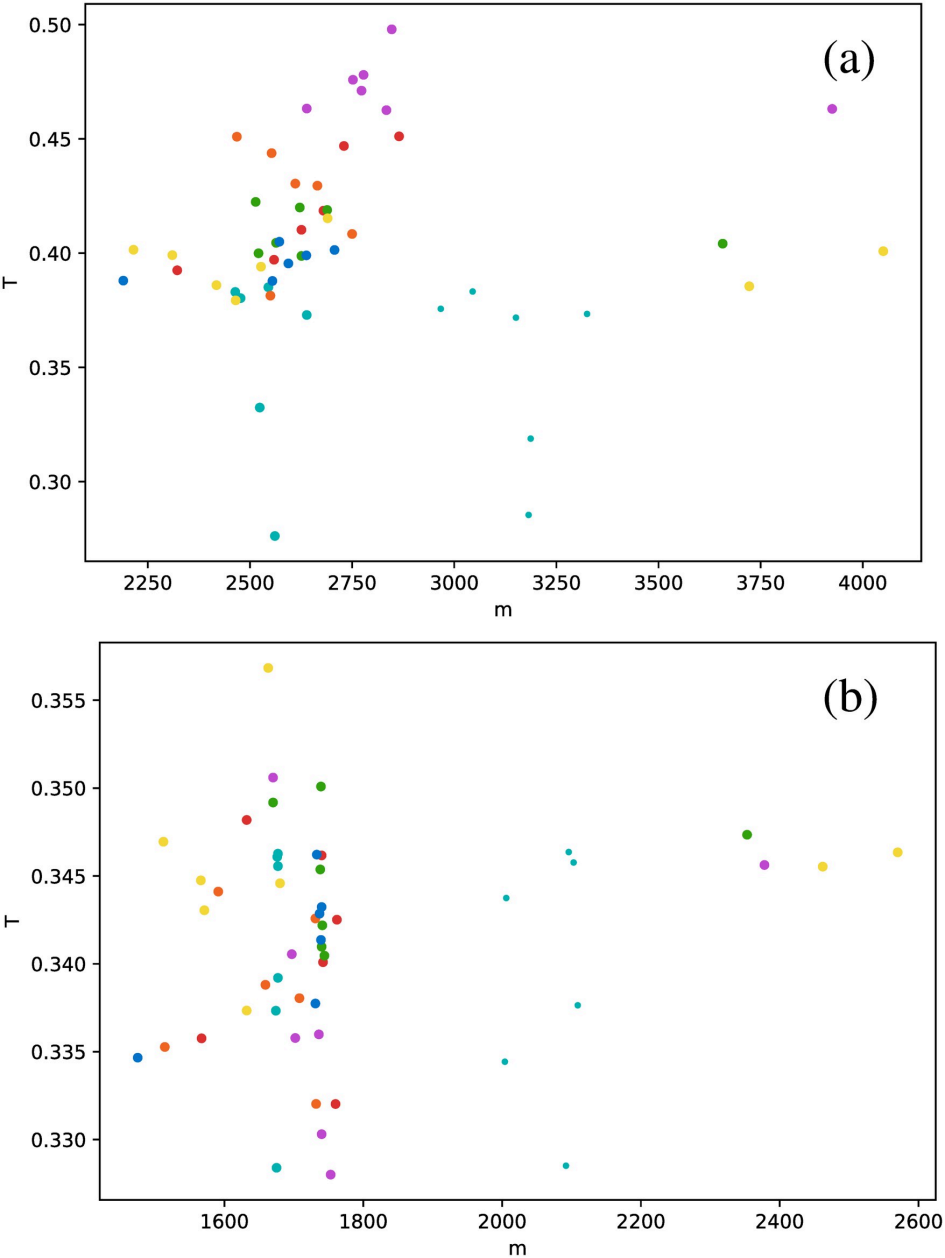
Same as Fig 6, but for the transitivity coefficient.

Calculation of the transitivity coefficient *T* highlights the nontriviality of these results. In principle, this metric is similar to the clustering coefficient because it measures the probability that a triad in the network is a triangle. However, for the case of the studied stars, markedly different results are obtained, as shown in Fig 8. Unlike in Figs 6 and 7, there is no special behavior of *δ* Scuti and RR Lyrae RRd stars in the HVG. The HVG thus does not seem to discriminate between star types. However, results are different for the VG: Cepheids are in the fundamental mode (magenta dots), RR Lyrae RRab (green dots), and RR Lyrae RRd stars (blue dots) are confined to a much narrower range of values than the other star types.

When we discussed Fig 7 above, we mentioned that, in general, there is no correlation between the clustering coefficient and the degree for a node, or for their average values over a network, and thus are independent metrics for an arbitrary network.

**Fig 9:**
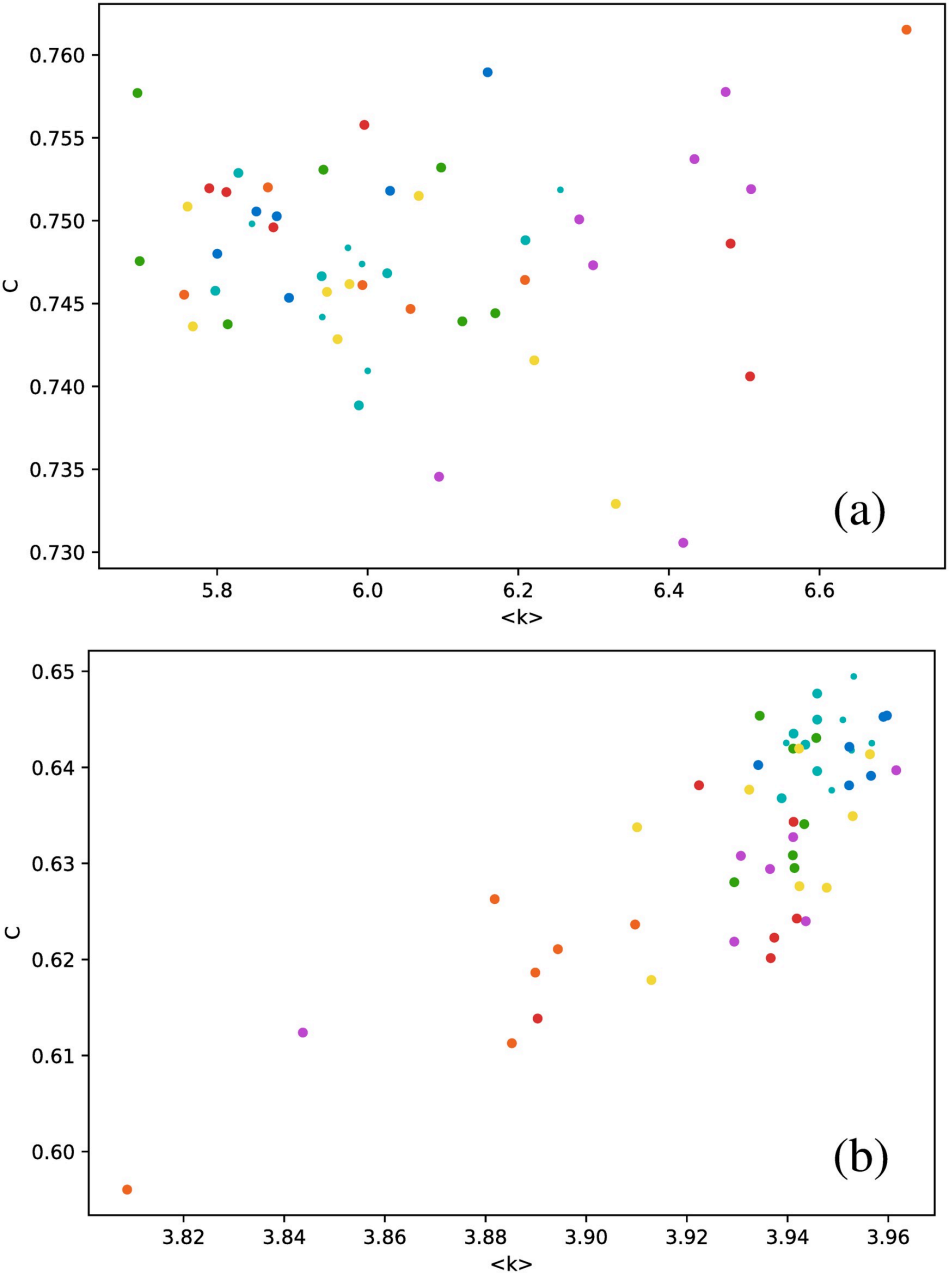
Same as Fig 6, but for the clustering coefficient as a function of the average degree.

Despite this, it is again interesting to notice that, for the light curves studied, there is an approximate correlation between the average clustering coefficient and the average degree, as shown in Fig 9, which suggests an approximately linear correlation between these metrics for the HVG. No such result is observed for the normal VG.

**Fig 10:**
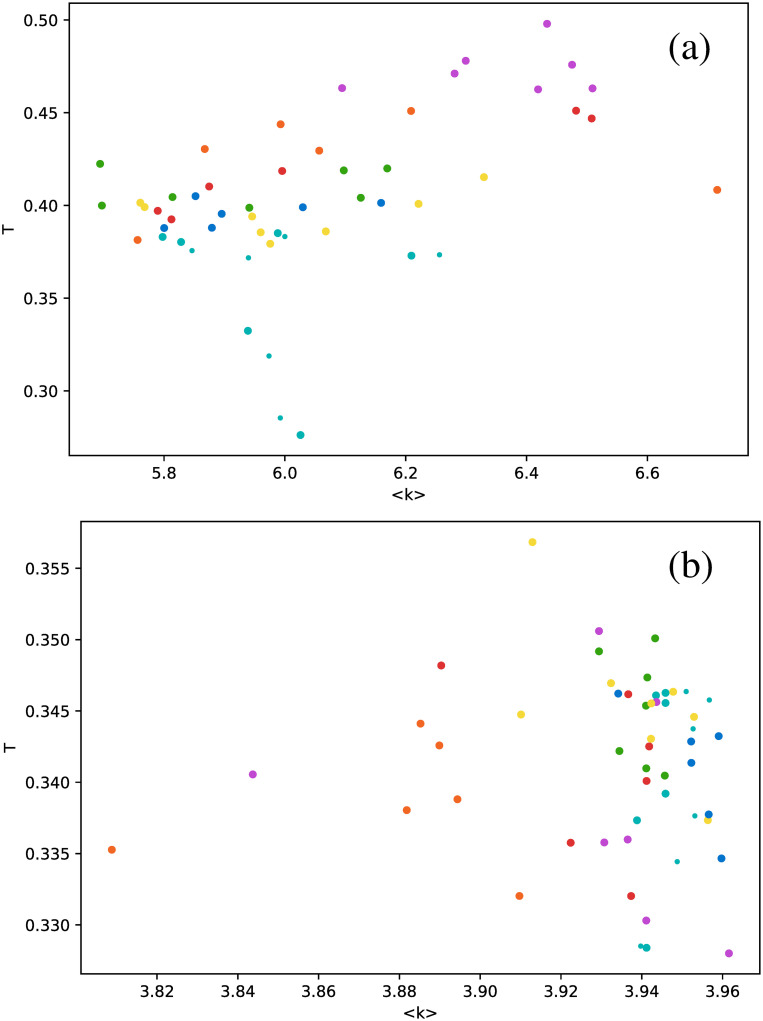
Same as Fig 6, but for the transitivity coefficient as a function of the average degree.

As another example of the differences between seemingly similar metrics, no correlation is found for the transitivity coefficient, as shown in Fig 10.

These results have been obtained for the full time series, including the eventual gaps. To investigate the possible effect of gaps in the findings, we perform the same analyses but for each observation window (Observation Windows Section) and for phased light curves (Phased light curves Section).

### Observation windows

Now, we split each time series into windows separated by the observation gaps. For each window, we calculate the same metrics discussed in the Full time series section, and we average over each set of windows to calculate a representative value for each star.

This method allows us to eliminate the effect of gaps, which is a feature of the time series that is not related to the variability of the star itself, and thus we should expect to have “better” results that are more representative of the star itself. However, the cost is that time series are much shorter (approximately 200 points), and the statistics for each network are markedly worse. Thus, whatever results we obtain should be considered from this perspective.

**Fig 11:**
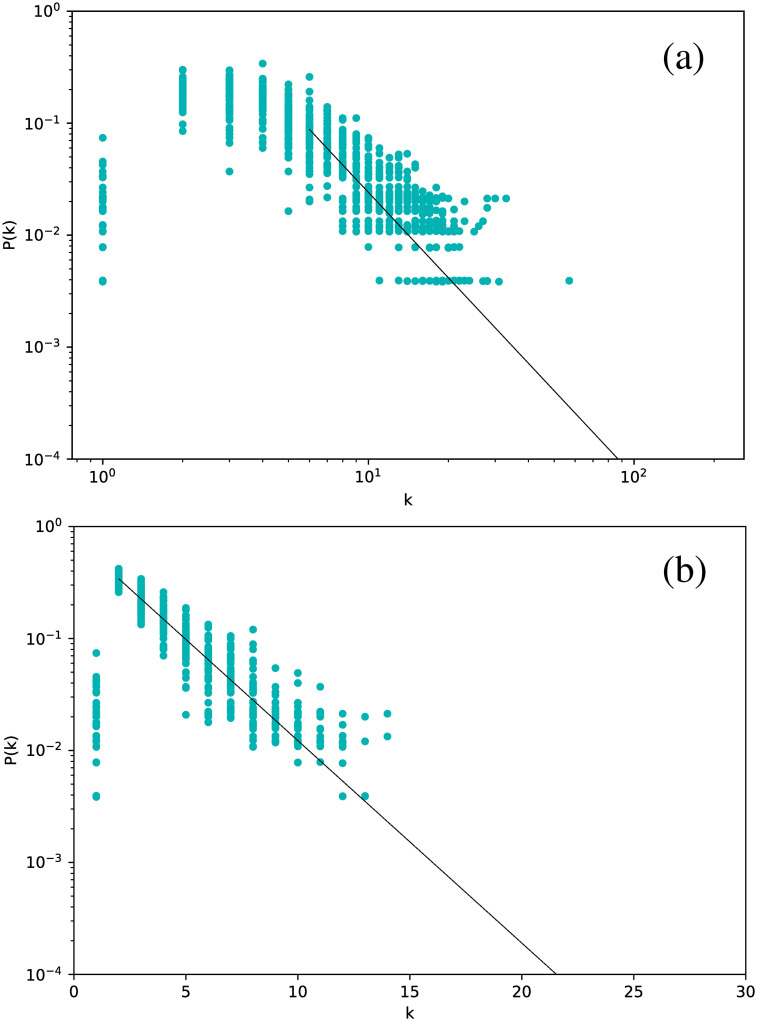
Same as Fig 2, but averaging over networks built from each observation window for each star.

Although this strategy leads to smaller networks, we still obtain meaningful degree distributions, as shown in Fig 11, which gathers the results for all six *δ* Scuti stars.

Fig 11 shows that there is no major difference in the distributions when the data are split into observation windows, or when the full time series is considered, including its gaps (Fig 2): we still observe a power-law decay for the VG and an exponential decay for the HVG. Thus, these results look like robust features that are not affected by data gaps. As previously mentioned, one would expect that the HVG would be less sensitive to the gaps than the VG, but we observe in this study that, at least regarding the degree distribution, the VG is also insensitive to them.

In a more detailed analysis, there are some differences between Figs 2 and 11. For both plots, corresponding to both VG and HVG, respectively, the range in the horizontal axis is now smaller due to the smaller size of the networks, which naturally leads to a smaller number of connections between nodes. Also, in the HVG case, a break in the power law is found for smaller degrees, which may also be due to the worse statistics [31].

Similar results are found for all star types. Table 3 shows the best fit for the tail of the degree distribution. As in Table 2, the exponent for the HVG corresponds to an exponential fit and for the VG to a power-law fit. For *δ* Scuti stars, only the results for the truncated time series were used. Within the first 850 points, 11 observation windows are contained, which is sufficient to compare results with the rest of the stars. (The full time series would comprise 13 windows; thus, the difference is not significant.)

**Table 3:** Same as Table 2, but for averages over all observation windows.

Star Type	*γ*_HVG_	*γ*_VG_
*δ* Scuti (t)	0.416 ± 0.004	2.53 ± 0.02
Cepheids F	0.415 ± 0.005	2.52 ± 0.02
Cepheids 1O	0.418 ± 0.005	2.54 ± 0.02
Cepheids 1O/2O	0.420 ± 0.004	2.47 ± 0.02
RR Lyrae RRab	0.408 ± 0.004	2.61 ± 0.02
RR Lyrae RRc	0.407 ± 0.004	2.46 ± 0.02
RR Lyrae RRd	0.415 ± 0.004	2.44 ± 0.02

In the first order, results are consistent with those in the Full time series Section when the full time series is used: *γ*_HVG_ ∼ 0.4 and *γ*_VG_ ∼ 2.5. However, in this case, the HVG can discriminate better between star types than it did for the full time series, and the exponent for *δ* Scuti stars is not clearly higher than the rest. In general, *γ*_HVG_ marginally increases with respect to Table 2 for all star types. Also, RRab and RRc stars have appreciably lower exponents than the other stars.

Regarding the VG results, discrimination between star types is maintained, but no particular trend can be found when compared with Table 2. In general, *δ* Scuti and Cepheid stars have larger exponents than RR Lyrae stars, with the exception of Cepheids 1O/2O and RR Lyrae RRab; thus, this statement is less robust than Table 2.

Thus, qualitative results for the shape of the degree distribution are robust, regardless of using full time series or individual observation windows, even the quantitative results are similar to the first order. However, when examined in more detail, the HVG and VG change differently. Most notably, the HVG seems to improve its discrimination capabilities marginally when smaller timescales are used.

**Fig 12:**
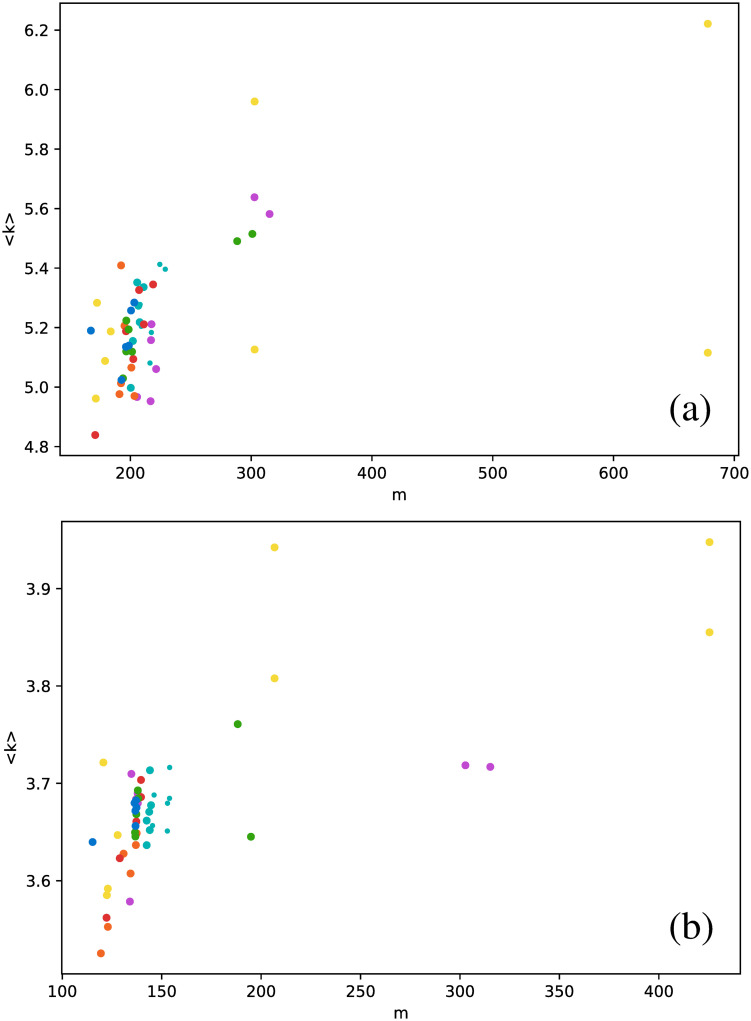
Same as Fig 6, but for the average degree as a function of the number of connections, and averaging over networks built from each observation window for each star.

**Fig 13:**
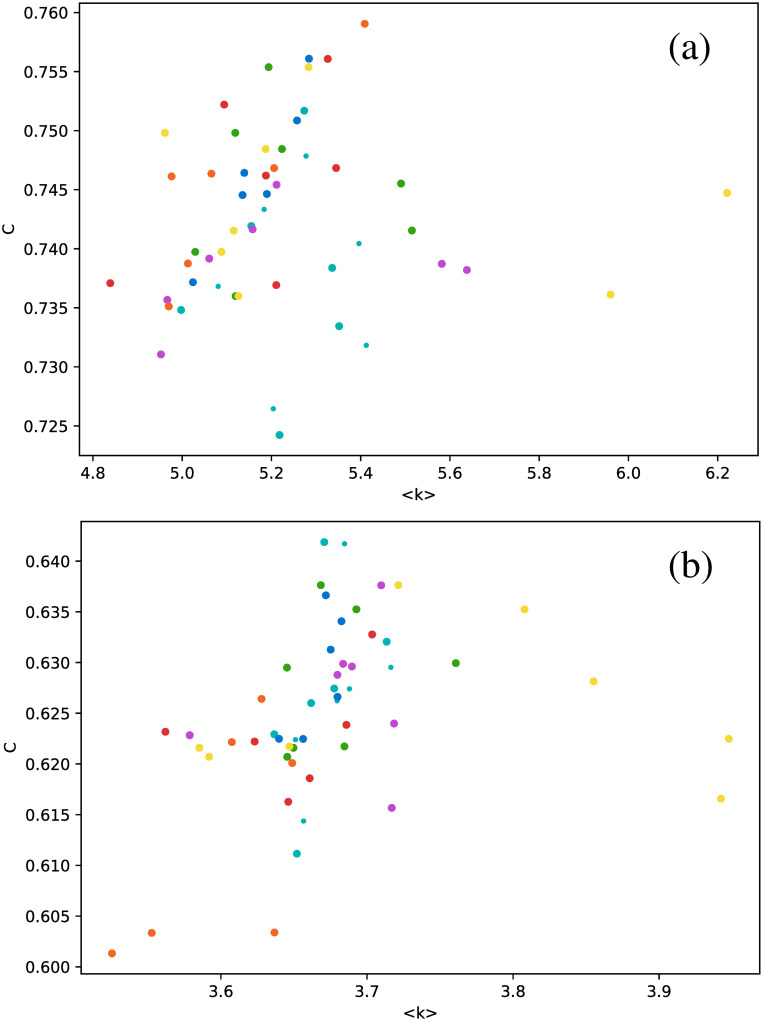
Same as Fig 12, but for the clustering coefficient.

**Fig 14:**
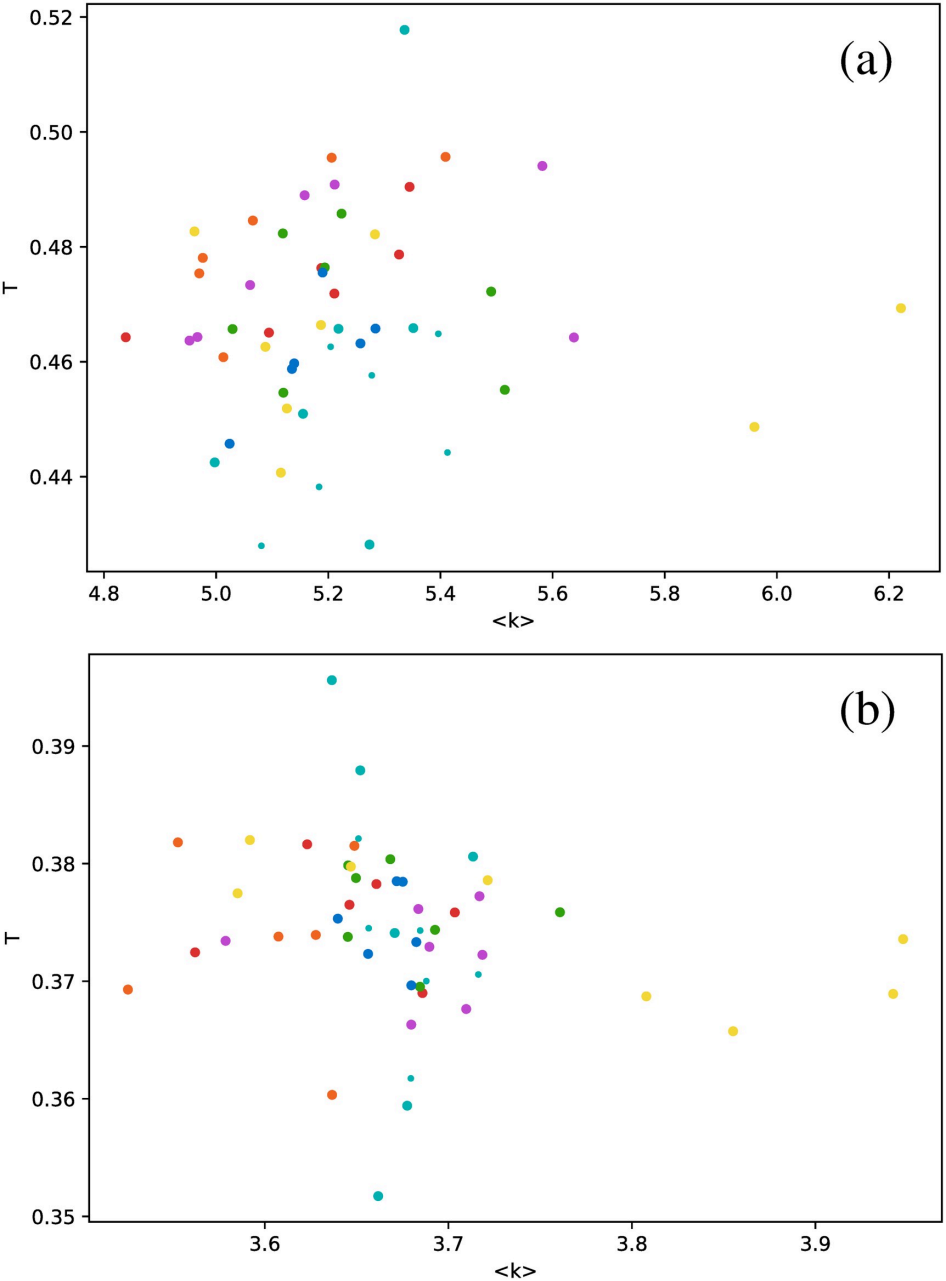
Same as Fig 12, but for the transitivity coefficient.

When the average degree is calculated for each star, we obtain Fig 12. All *δ* Scuti stars are essentially in the same abscissa, due to the selection of the first 850 data points mentioned in Full time series Section. As before, we focus on the spread of points in the vertical axis. RR Lyrae RRd (blue dots) and RR Lyrae RRab (green dots) are strongly concentrated in a small band for 〈*k*〉. We have also observed a strong concentration of these stars in some previous metrics when the full time series was taken (Full time series Section). The same phenomenon also can be observed for the clustering coefficient (Fig 13) and transitivity coefficient (Fig 14) for the same two types of stars. It is not as a strong grouping as for the average degree, but these types of stars tend to cover a narrower band than the other stars.

It is also interesting that this particular feature is observed, more or less clearly, for both types of visibility graphs. When the full time series is used, these trends appeared for either the normal VG or the HVG.

We also notice the opposite behavior in *δ* Scuti stars, as with all three metrics (Figs 12–14), they tend to cover a wider range of values than all the other star types.

### Phased light curves

Our last method to build complex networks uses phased light curves. The period required to create a phased curve was taken from the information provided by the OGLE-III catalog itself.

**Fig 15:**
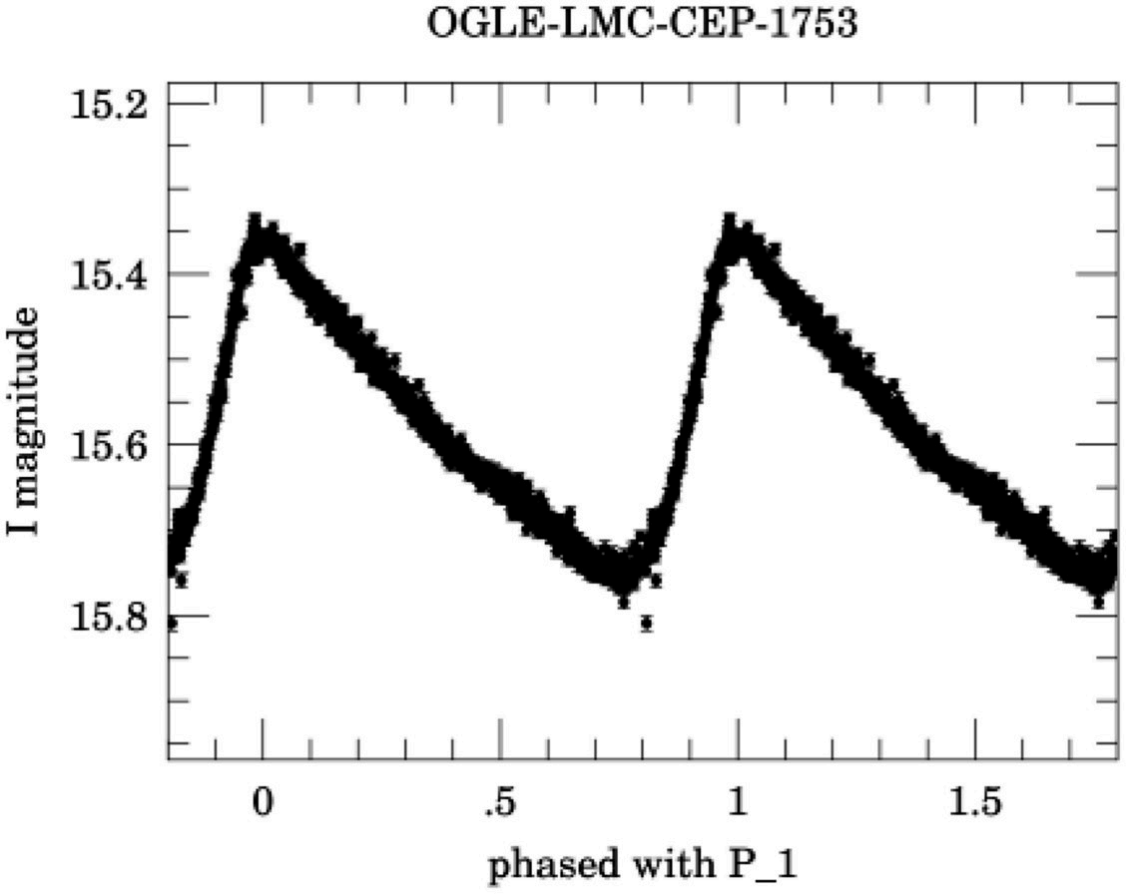
Phased light curve of star OGLE-LMC-CEP-1753, as given by the OGLE-III catalog [27].

A typical phased curve is shown in Fig 15. The phased curve involves a drastic change in the geometry of the time series (gaps are filled, and the periodicity is lost because, by definition, the light curve spans a single period). Therefore, considering that it is such a valuable tool to describe the evolution of luminosity in variable stars, it is interesting to see how it modifies the results when the visibility graph technique is applied to it.

**Fig 16:**
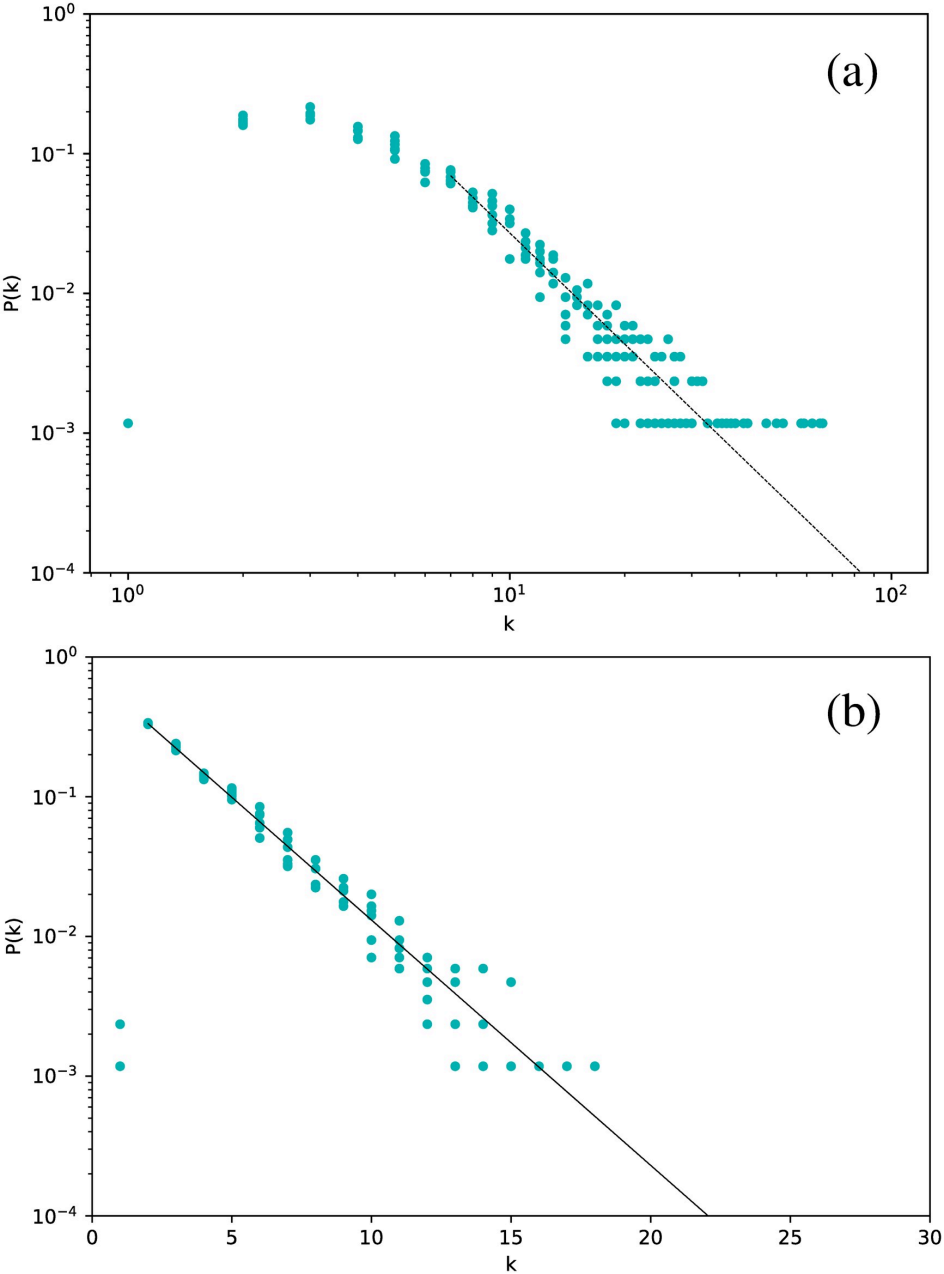
Same as Fig 2, but for phased light curves of *δ* Scuti stars.

The qualitative results obtained thus far are not affected by using phased curves, as shown in Fig 16, where the degree distribution for all *δ* Scuti stars in Table 1 are plotted on a semilog scale for the HVG, and a log-log scale for the VG. An exponential behavior is found for the HVG, and a power-law behavior for the VG, which are consistent with the results obtained when the original time series is used (Full time series section), and when the time series is split in observation windows (Observation windows Section).

**Fig 17:**
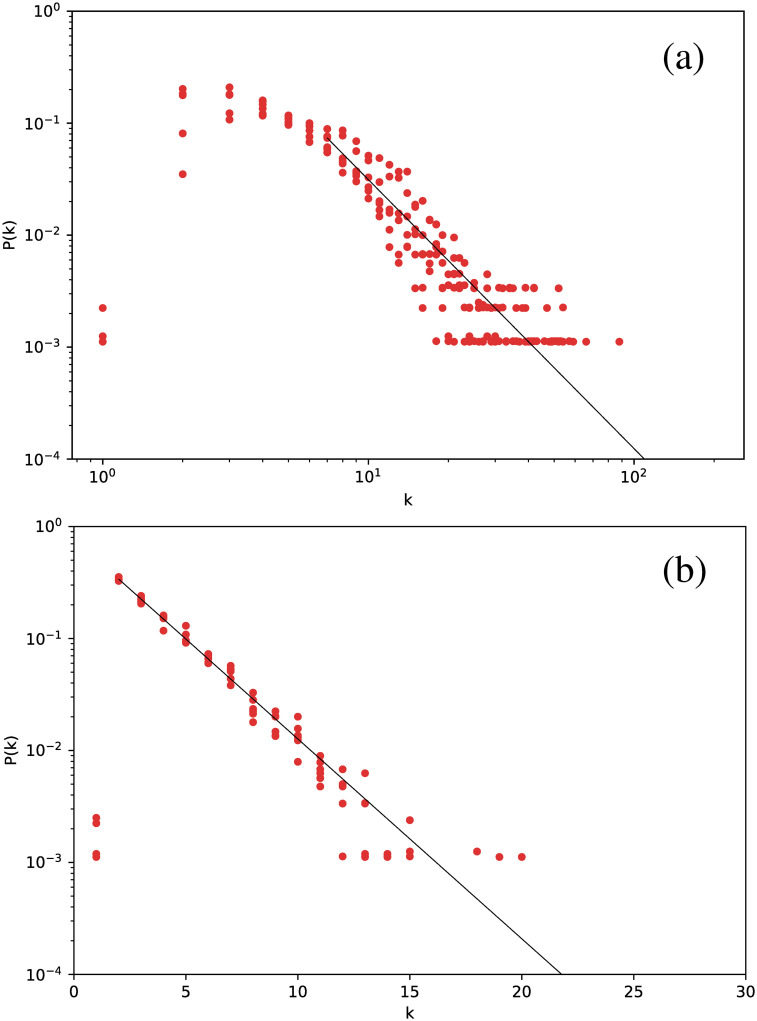
Same as Fig 2, but for phased light curves of Cepheids pulsating in two modes.

Fig 17 shows the same plot but for Cepheids pulsating in two modes and phased with the period *P*_1_, where the same trend is found again.

Similar results are found for all star types. Table 4 shows the best fit for the tail of the degree distribution, an exponential fit for the HVG and a power-law fit for the VG. To build the phased light curves, the period of the star must be known. Thus, in the case of stars that oscillate in two modes (Cepheids 1O/2O, RR Lyrae RRd, and RR Lyrae RRd), two phased lights were used, each corresponding to the period of one of the modes; this fact is indicated by *P*_1_ and *P*_2_ in Table 4. With *δ* Scuti stars, the truncated case shows that, the first 850 points were taken, and the resulting time series was phased.

**Table 4:** Same as Table 2, but for the phased light curves.

Star Type	*γ*_HVG_	*γ*_VG_
*δ* Scuti	0.411 ± 0.004	2.44 ± 0.04
*δ* Scuti (t)	0.405 ± 0.003	2.64 ± 0.03
Cepheids F	0.437 ± 0.005	2.63 ± 0.06
Cepheids 1O	0.425 ± 0.006	2.44 ± 0.02
Cepheids 1O/2O (*P*_1_)	0.411 ± 0.004	2.40 ± 0.04
Cepheids 1O/2O (*P*_2_)	0.404 ± 0.003	2.46 ± 0.02
RR Lyrae RRab	0.408 ± 0.004	2.38 ± 0.02
RR Lyrae RRc	0.406 ± 0.003	2.44 ± 0.02
RR Lyrae RRd (*P*_1_)	0.405 ± 0.004	2.46 ± 0.02
RR Lyrae RRd (*P*_2_)	0.405 ± 0.003	2.65 ± 0.03

The values in Table 4 are similar to those in previous ones to the first order, which allows us to further establish the robustness of the results. When examined in more detail, the HVG exponents are lower for RR Lyrae than for *δ* Scuti and Cepheids, a feature that we already found in the full time series and observation window sections, and thus also seems to be a robust feature. The VG exponent is also lower for RR Lyrae, although Cepheids 1O/2O and RR Lyrae RRd are exceptions to this trend. Note that Cepheids 1O/2O are also an exception to this when observation windows are used (Table 3); thus, this could be a result worth exploring more systematically in future research.

**Fig 18:**
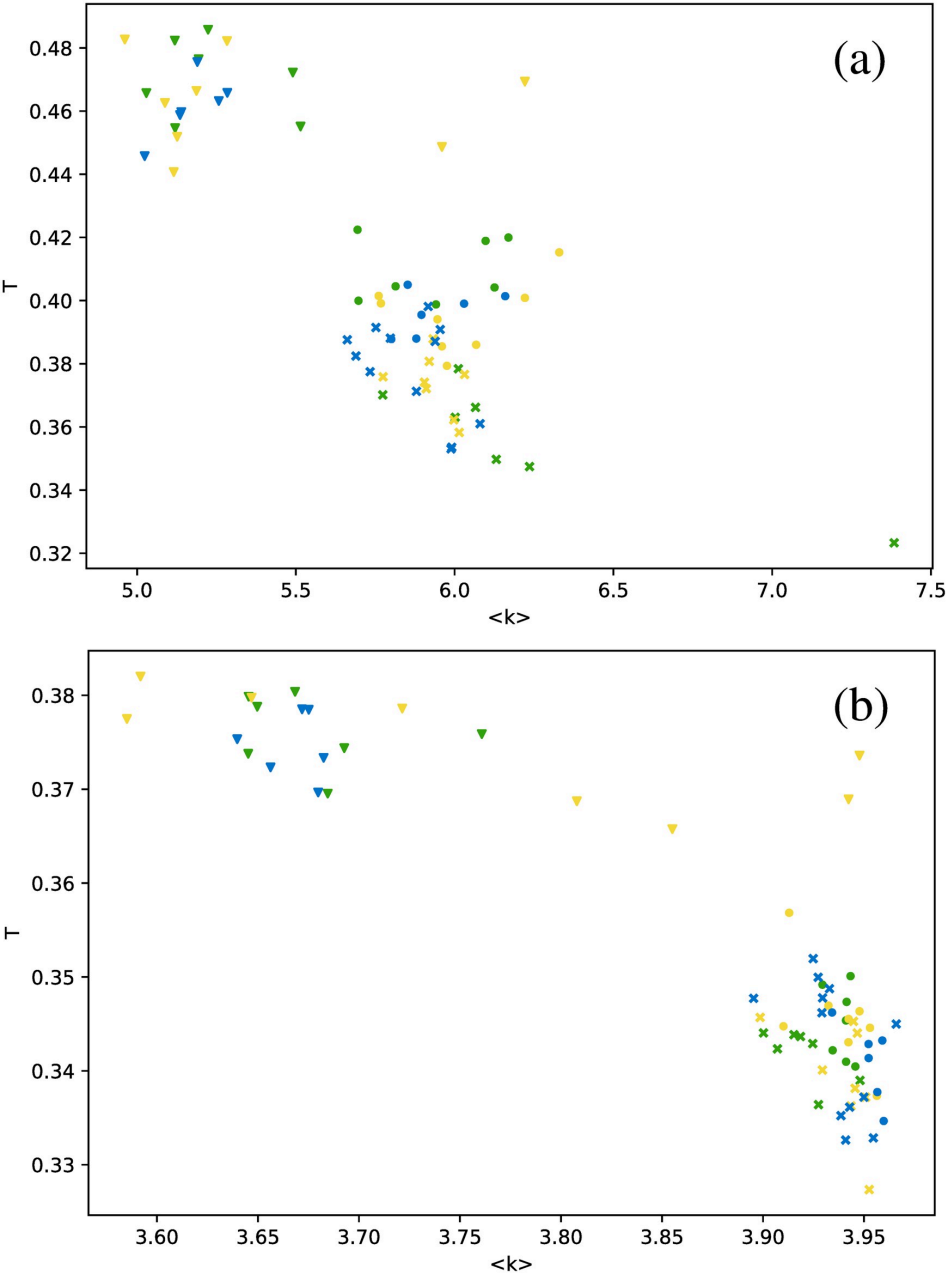
Transitivity as a function of average degree for the three types of RR Lyrae stars. Circles: full time series results; triangles: observation windows; crosses: phased light curves.

**Fig 19:**
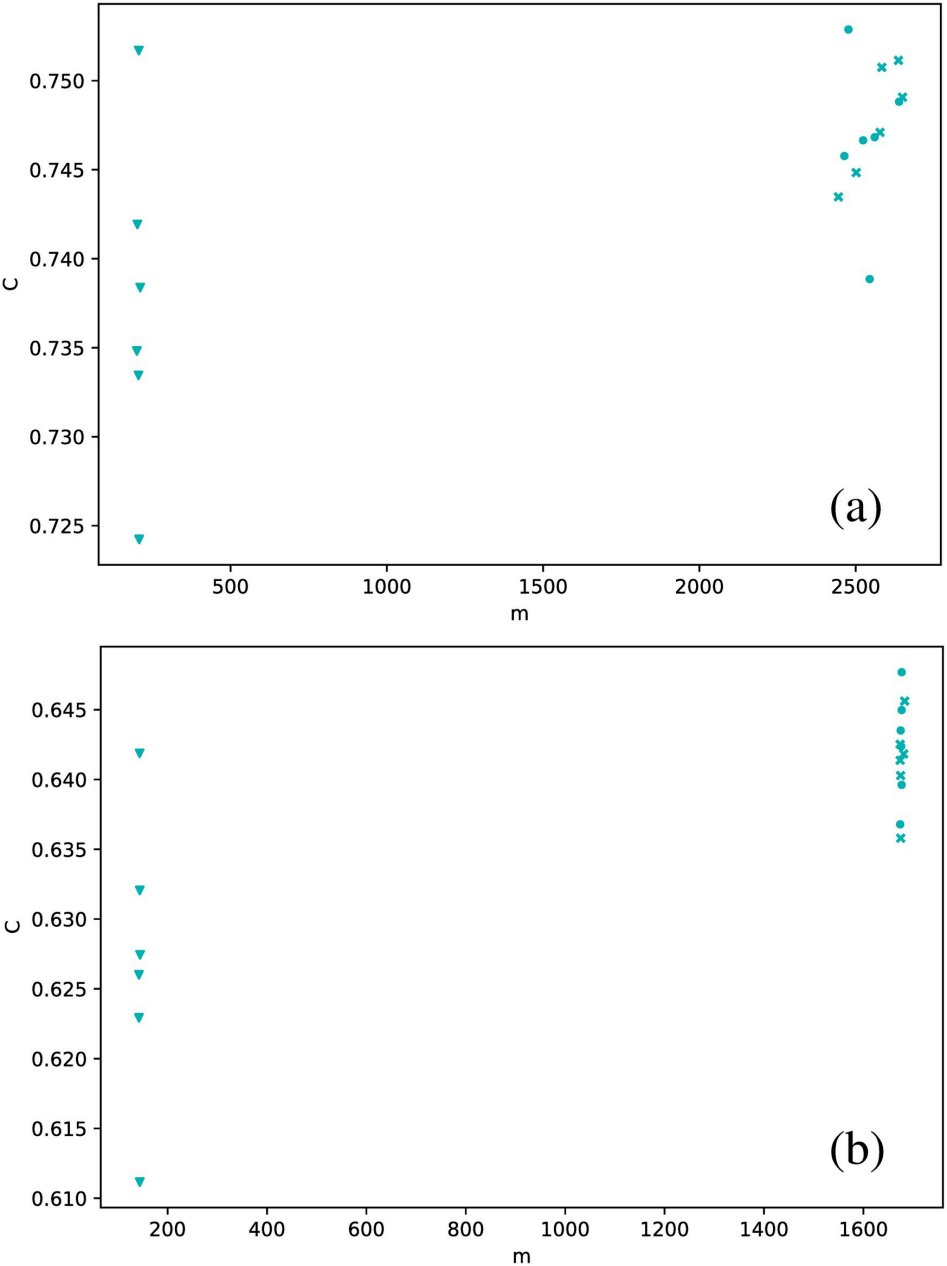
Same as Fig 18, but for clustering coefficient as a function of the number of connections, for the *δ* Scuti stars.

An interesting result emerges when the correlation between metrics is explored for all three strategies to manage the time series (full time series, observation windows, and phased time series). Values obtained for full and phased light curves are much more similar to each other than to the results for observation windows; this is shown in Fig 18 for the average degree versus transitivity in RR Lyrae stars, and Fig 19 for the number of connections versus clustering coefficient in *δ* Scuti stars. In both cases, results for full time series (dots) and phased time series (crosses) cluster in a separate cloud, apart from the results from observation windows (triangles).

Similar results are obtained for nearly all possible pairs of metrics studied in this paper, which is remarkable because, as mentioned before, the geometry of the phased curve is markedly different from the full light curve, and yet the (H)VG seems to capture the same structural properties. The different results found with the observation windows may be due to size effects in the time series, but as shown in the Observation Windows Section, the findings are consistent within the various star types, despite the worse statistics.

**Fig 20:**
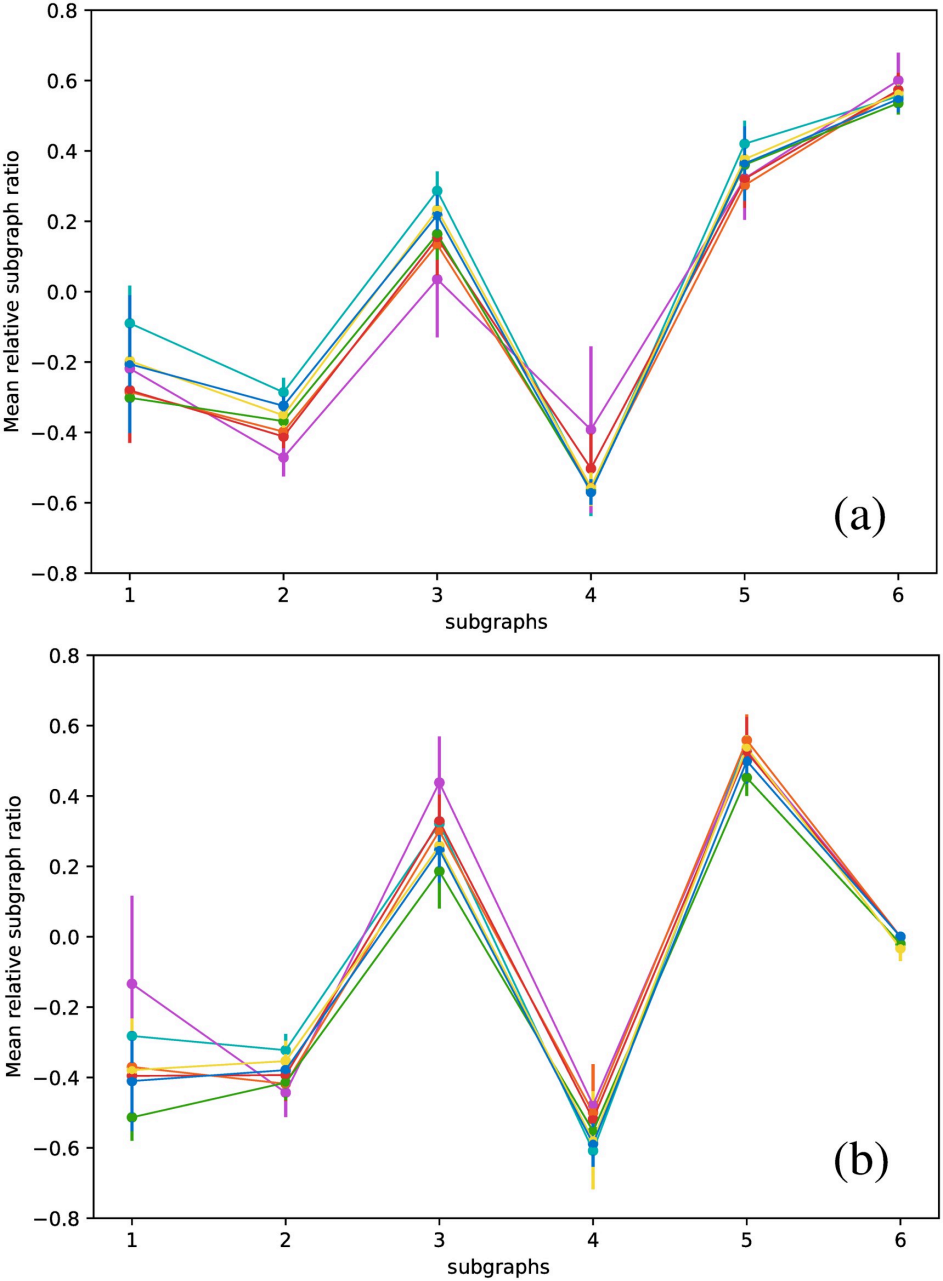
Mean relative subgraph ratio as a function of the type of subgraph. (a) VG, (b) HVG.

Note that all of these results are based on metrics that describe global features of the networks. Other alternatives are possible. For example, we could study the statistics of subgraphs within the network, as proposed in [32], which shows that six basic subgraphs can discriminate between various network types. We have used the same technique for the variable stars studied in this study; however, as shown in Fig 20, all star types yield essentially the same results, both for the VG and the HVG graphs. This result suggests that such analysis of local features cannot discriminate between types of pulsating stars, at least if the (H)VG techique is used, and that global methods such as those we present in this study may be a more useful approach.

## Conclusion

We have used the visibility graph (VG) algorithm to study light curves of variable stars. We have focused on six types of stars, which include various modes of oscillations (purely radial, radial and nonradial, fundamental mode or overtones), to study to what extent the technique is able to uncover universal features or to discriminate between types of stars.

Thus, we used two versions of the visibility graph algorithm: the normal visibility graph (VG) and the horizontal visibility graph (HVG). We have also used three versions of the light curves: the full time series, including all its observation gaps; the time series split into observation windows; and the phased light curve. Finally, we have studied three metrics for each network: the degree (its distribution and its average), the clustering coefficient and the transitivity coefficient.

Several robust findings emerged from these analyses. In all cases, the normal visibility graph leads to power-law degree distributions, while the horizontal visibility graph leads to exponential degree distributions. Decay exponents are similar for all star types, but a marginal difference is observed for *δ* Scuti stars.

Thus, in general, we find that the degree distribution seems to be essentially the same for all types of stars rather than discriminating between types. We should stress that, for an arbitrary network, degree distributions are not necessarily exponential or scale-free; thus, this result tells us something about the light curves themselves. In general, exponential degree distributions may be associated with fully random networks, while scale-free networks can be attributed to the existence of preferred nodes. However, what features must have a time series to be mapped to such networks is a far from trivial matter. For example, for fully uncorrelated chaotic or correlated stochastic time series, the HVG has an exponential degree distribution [23]. However, light curves do not belong to these categories, and thus it would be interesting to study whether the noise level is so high that the HVG cannot distinguish them from uncorrelated chaotic or correlated stochastic time series; or whether the geometry of the pulsating time series leads to such results. Work on simulated time series could help resolve this issue.

When specific metrics (e.g., average degree, clustering coefficient, transitivity coefficient) are considered, differences between star types emerge. For some star types and graphs, the metrics lie in a small range of values (e.g., for Cepheids pulsating in the fundamental mode, RR Lyrae RRab, RR Lyrae RRc), while other star types span a wider range for all metrics (*e.g*. *δ* Scuti stars, when the networks were built for each observation window). Thus, although the VG algorithm does not seem to allow complete discrimination between variable star types, it does offer an opportunity for at least some of them.

Considering that the various metrics are independent quantities, it is interesting that some stars (e.g., RR Lyrae RRab and RRc, and delta Scuti) exhibit a similar behavior when studied under different metrics.

However, one correlation that did appear is the nearly linear behavior of the clustering coefficient as a function of the average degree with the full time series (Fig 9). As already mentioned, metrics are independent values; thus, this result is an intriguing finding that deserves further investigation.

It is also interesting to notice the robustness of the (H) VG method as a function of the gaps in the time series. As shown in Figs 4 and 16, the full time series with all its observation gaps yields results that are consistent with the phased time series. Even if one takes each observation window and analyzes them separately, the results are qualitatively similar, as observed, for example, in the degree distribution functions for each graph (Observation windows Section).

We find that the visibility algorithm is a useful way to study the light curves of variable stars, showing interesting features, some of them universal, for all the stars studied, while others seem to discriminate between such types. It is also interesting that this method does not seem to be affected by the three different ways to deal with the gaps, meaning that the difficult problem of resolving gap existence is not too important for this construction, and that we can obtain valuable information from these light curves regardless of observation gaps.

To our knowledge, this is the first study to apply the VG algorithm to the problem of star variability; thus, there is more research to be performed to better understand these results. We are already studying simulated time series, which may help to distinguish between accidental and robust results systematically. The possibility to train a neural network to discriminate between variable star types would be an interesting consequence of the results in this paper, but more variable stars should be analyzed to improve statistics. Another point of interest is to calculate other metrics that should provide information not covered by those considered in this paper. These issues will be considered in future publications.
